# Review of Preclinical and Clinical Studies of Bone Marrow-Derived Cell Therapies for Intracerebral Hemorrhage

**DOI:** 10.1155/2016/4617983

**Published:** 2016-09-06

**Authors:** Paulo Henrique Rosado-de-Castro, Felipe Gonçalves de Carvalho, Gabriel Rodriguez de Freitas, Rosalia Mendez-Otero, Pedro Moreno Pimentel-Coelho

**Affiliations:** ^1^Instituto de Ciências Biomédicas, Universidade Federal do Rio de Janeiro, 21941-902 Rio de Janeiro, RJ, Brazil; ^2^Departamento de Radiologia, Universidade Federal do Rio de Janeiro, 21941-902 Rio de Janeiro, RJ, Brazil; ^3^Departamento de Neurocirurgia, Universidade do Estado do Rio de Janeiro, 20551-030 Rio de Janeiro, RJ, Brazil; ^4^Instituto Estadual do Cérebro Paulo Niemeyer, 20231-092 Rio de Janeiro, RJ, Brazil; ^5^Instituto D'Or de Pesquisa e Ensino, 22281-100 Rio de Janeiro, RJ, Brazil; ^6^Instituto de Biofísica Carlos Chagas Filho, Universidade Federal do Rio de Janeiro, 21941-902 Rio de Janeiro, RJ, Brazil

## Abstract

Stroke is the second leading cause of mortality worldwide, causing millions of deaths annually, and is also a major cause of disability-adjusted life years. Hemorrhagic stroke accounts for approximately 10 to 27% of all cases and has a fatality rate of about 50% in the first 30 days, with limited treatment possibilities. In the past two decades, the therapeutic potential of bone marrow-derived cells (particularly mesenchymal stem cells and mononuclear cells) has been intensively investigated in preclinical models of different neurological diseases, including models of intracerebral hemorrhage and subarachnoid hemorrhage. More recently, clinical studies, most of them small, unblinded, and nonrandomized, have suggested that the therapy with bone marrow-derived cells is safe and feasible in patients with ischemic or hemorrhagic stroke. This review discusses the available evidence on the use of bone marrow-derived cells to treat hemorrhagic strokes. Distinctive properties of animal studies are analyzed, including study design, cell dose, administration route, therapeutic time window, and possible mechanisms of action. Furthermore, clinical trials are also reviewed and discussed, with the objective of improving future studies in the field.

## 1. Introduction

Stroke is the second cause of death in the world, behind ischemic heart disease [1]. About 10 to 27% of strokes are hemorrhagic in presentation [2]. It has been estimated that in 2013 there were a total of 6.5 million deaths due to stroke (49% from hemorrhagic strokes) [3] and that stroke was the second greatest cause of disability-adjusted life years (DALYs), causing 113 million DALYs (42% due to hemorrhagic strokes) [3].

Intracerebral hemorrhage (ICH) can be classified as primary or secondary, depending on the cause of the hemorrhage. Primary ICH is caused by a spontaneous rupture of small arteries by chronic hypertension or amyloid angiopathy. In secondary ICH, the hemorrhage is caused, for example, by trauma, vasculopathies, coagulopathies, and tumors, among other causes [4].

Unlike the ischemic infarct, which often has an acute onset, ICH usually has a progressive onset. Symptoms vary according to the location and size of the hematoma [5]. In 40% of the cases, the patient will have intraventricular hemorrhage, which is associated with a worse outcome and with an increased risk of developing hydrocephalus [4]. Delayed deterioration is not uncommon and is usually due to rebleeding, edema, hydrocephalus, or seizures.

Many studies have been trying to elucidate the best treatment option for this catastrophic pathology since there are still many controversies regarding the management of patients with ICH. For this reason, every three years the American Heart Association and the American Stroke Association perform a review of the literature and elaborate an evidence-based guideline for the management of ICH [6].

Despite all efforts, the overall 30-day mortality rate of ICH patients can reach 40–50% [7]. Although a large number of randomized medical trials have been completed, they all failed to prove any benefit of different drugs or surgical interventions in patients with ICH [8, 9]. Current treatment is based on the prevention of secondary brain injury, including rebleeding and secondary brain ischemia. General measures, such as the control of blood pressure, reduction of intracranial pressure (ICP), and prevention of infections, are important to prevent secondary brain injuries [7]. Indeed, the presence of secondary clinical complications, such as pneumonia, brain edema, cardiac complications, and sepsis, was correlated with a higher mortality in surgically and conservatively treated patients [10].

The general practice is to operate on young patients with large superficial lobar hematomas with mass effect and that are leading to an uncontrolled increase in ICP. In addition, posterior fossa hematomas are usually surgically evacuated, when they are larger than 3 cm in diameter and are causing a mass effect on the brainstem. In summary, each medical decision should be individualized based on patient's neurosurgical condition, size and location of hematoma, patient's age, and family wishes. Normally, ICHs are evacuated through a regular craniotomy, with or without the use of a microscope. New types of surgery have been studied and tried, including minimally invasive techniques with smaller incisions. However there is a lack of evidence that those new techniques are more efficient than the regular craniotomy [6, 11].

In this scenario, cell-based therapies represent a promising approach for the treatment of hemorrhagic stroke. Accumulating evidence suggests that different types of stem cells have the potential to induce or accelerate functional recovery in animal models of ICH and subarachnoid hemorrhage (SAH) [12–14]. As described by Hu et al. [12], mesenchymal stem cells (MSC) and neural stem cells were the most frequent cell types investigated in these studies. Neural stem cells are defined by their capacity to self-renew and give rise to neurons and glial cells. They can be derived from embryonic stem cells and induced pluripotent stem cells or can be isolated from fetal and adult neurogenic niches [15]. In contrast, MSC are plastic adherent stromal cells, characterized by their ability to self-renew and differentiate into osteocytes, chondrocytes, and adipocytes, but not into cells of nonmesodermal origin. MSC are present in the bone marrow and in numerous other fetal and adult tissues, including the umbilical cord, placenta, and adipose tissue, from which they can be isolated and expanded in culture [16, 17]. Furthermore, MSC can be generated from embryonic stem cells and induced pluripotent stem cells [18, 19].

In view of the increasing use of bone marrow-derived MSC (BM-MSC) in clinical trials for ischemic and hemorrhagic strokes [20], the aim of this review is to discuss current evidence from preclinical and clinical studies that have investigated the therapeutic potential of BM-MSC in ICH. Because the bone marrow contains other cell types with potential therapeutic value in ICH, including lymphocytes, monocytes, endothelial progenitor cells, and hematopoietic stem/progenitor cells, which compose the mononuclear cell fraction [21], studies that have used bone marrow mononuclear cells (BM-MNC) were included in the present review.

## 2. Preclinical Studies

**Figure 1:**
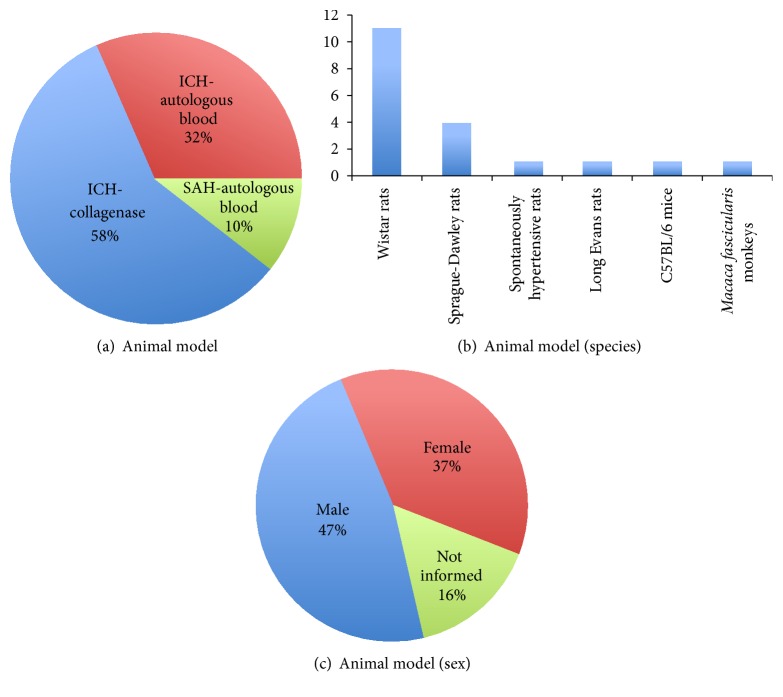
Charts summarizing the animal models (a), species (b), and sex of the animals (c) used in preclinical studies that have evaluated the potential of bone marrow-derived cells to treat hemorrhagic stroke.

We searched the PubMed/Medline database for original articles in English that have evaluated the therapeutic role of bone marrow-derived cells in animal models of ICH and SAH. We identified 18 articles in which BM-MSC were transplanted and 1 article in which BM-MNC were transplanted. The collagenase model of ICH was used by 11 studies while the remaining studies used the autologous blood model of ICH (6 studies) and SAH (2 studies) (Figure 1(a)). Almost all studies used rats, with the exception of two studies that used mice and monkeys (Figure 1(b)).

### 2.1. Cell Type, Dose, Administration Route, and Time Window for Therapy

**Figure 2:**
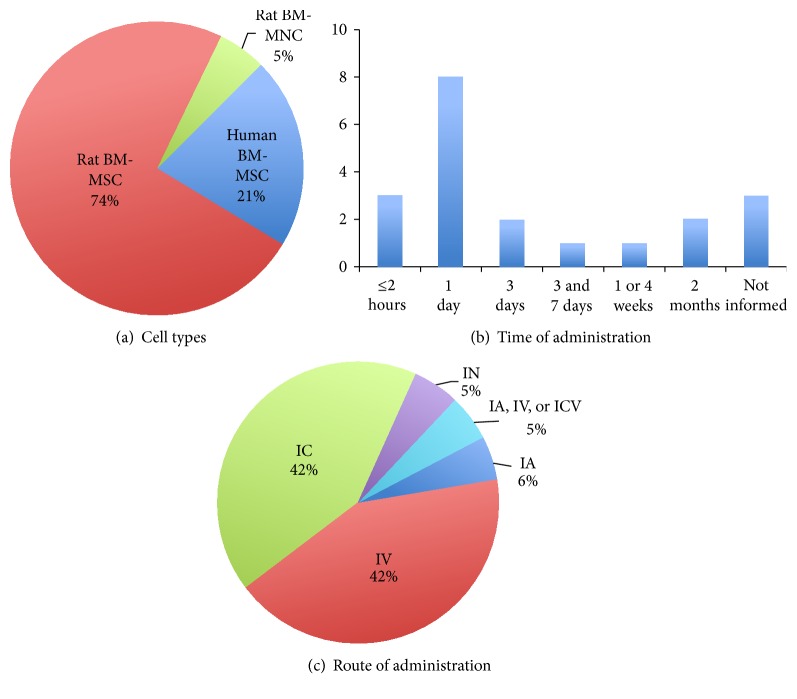
Charts summarizing the type of bone marrow-derived cells (a), time of injection (b), and route of administration (c) in preclinical studies for hemorrhagic stroke.

In 14 of the 19 studies, rat BM-MSC were used while 4 other studies used human BM-MSC (Figure 2(a)). The remaining study used rat BM-MNC and it was the only study that injected autologous cells. All studies found beneficial effects of the treatment on structural and/or functional outcomes, as summarized in Table 1.

Most of the studies that we identified (12 of 19 or 63,17%) have injected BM-MSC or BM-MNC within the first 24 hours after the injury (Figure 2(b)). This is consistent with the fact that these cells are expected to modulate early events in the pathophysiology of ICH and SAH, by counteracting neuronal cell death and limiting detrimental inflammatory responses [22–26]. For instance, in a series of 3 studies, Seyfried and colleagues evaluated the effects of human BM-MSC transplantation in a model of autologous blood injection into the rat* striatum* [27–29]. Their data showed that doses ranging from 1 × 10^6^ to 8 × 10^6^ of BM-MSC, intravenously injected 24 hours after ICH, were equally effective in reducing tissue loss and improving the neurological function of treated animals [27, 29]. In contrast, the lower dose of 0.5 × 10^6^ cells was not capable of inducing such protective effect [29].

They have also tested the intra-arterial route of injection, by injecting 1 × 10^6^ cells into the internal carotid artery. Interestingly, the beneficial effects of intra-arterially delivered BM-MSC were only observed when the animals were pretreated with an intravenous injection of mannitol. The authors proposed that mannitol facilitated the entry of BM-MSC in the perihematomal region, through its effects on the blood-brain barrier [28].

Different results were obtained by Zhang et al. [30], who compared the effects of 3 routes of rat BM-MSC administration on the motor recovery of rats subjected to a model of collagenase injection into the* striatum*. In their study, while the intra-arterial and intracerebroventricular routes were able to improve the motor dysfunction (in the beam balance test), the intravenous delivery of BM-MSC showed no effect. Major differences between this work and the studies of Seyfried [27–29] were the source of BM-MSC (rat versus human cells, resp.), the model of ICH, and the behavioral tests used, making it difficult to compare their results. However, all subsequent studies have shown that the intravenous administration of BM-MSC promotes neurological recovery in models of ICH [23, 25, 31] and SAH [22].

The intracerebral route was the second most common route of BM-MSC administration in ICH models (Figure 2(c)) and also has led to an improvement of the functional outcome [24, 32–36]. Finally, Sun and coworkers [37] have shown that the intranasal administration could represent a less invasive option for the delivery of BM-MSC to the brain following ICH. Their work was based on previous studies showing that intranasally injected rat BM-MSC can migrate from the nasal mucosa through the cribriform plate into the intact and injured rodent brain [38, 39]. In these studies, animals were pretreated with an intranasal injection of hyaluronidase 30 minutes before the application of cells, a protocol that facilitated the migration of BM-MSC to the brain [39].

Although the therapeutic window of BM-MSC administration for ICH and SAH has not been defined, there is evidence that the delayed treatment could still be effective. Vaquero and coworkers have shown that the IC administration of BM-MSC, 2 months after the induction of ICH, improved the functional outcome of treated rats [34, 35]. This favorable effect on the neurological recovery was potentiated by embedding the cells in a platelet-rich plasma (PRP) scaffold. Notably, donor cells could be found near the injury site, up to 6 months after transplantation, and the use of PRP-derived scaffolds increased the number of engrafted cells. Although the delayed treatment did not affect the lesion volume, it stimulated the proliferation of endogenous cells in the subventricular zone (SVZ), one of the neurogenic niches of the adult brain [35]. Furthermore, one interesting study found that the neurological deficits were attenuated by BM-MSC, regardless of whether the cells were intracerebrally transplanted at 1 or 4 weeks after the induction of ICH in* Macaca fascicularis* monkeys. However, early treatment led to a better metabolic recovery, as assessed by serial positron emission tomography (PET) scans with ^18^F-fluorodeoxyglucose (^18^F-FDG) [32].

The majority of these studies have shown that BM-MSC and BM-MNC migrated to the vicinity of the hemorrhagic lesion, regardless of the administration route. Several different techniques were employed to track the transplanted cells, but none of the studies have used noninvasive imaging techniques, such as bioluminescence imaging (BLI), fluorescence imaging (FLI), and radiopharmaceutical cell tracking (recently reviewed in [40]). The time point of the analysis varied among the studies, ranging from 6 hours to 6 months (Table 1), indicating that at least a fraction of the transplanted cells can survive for long periods in the brain.

### 2.2. Mechanisms of Action

Although it was initially hypothesized that BM-MSC and BM-MNC could differentiate into neural cells, this hypothesis has been challenged by many studies [17, 41–43]. Recent evidence supports the theory that the therapeutic action of bone marrow-derived cells is mediated by paracrine mechanisms, including the release of immune mediators, growth factors, and extracellular vesicles [44, 45]. The success of BM-MSC-based therapies is founded on the ability of BM-MSC to sense changes in the local environment and modify their secretome in response to alarmins [46] and pathogen-associated molecular patterns [47]. Thus, a great research effort has been made to characterize the secretome of BM-MSC under different stimulatory conditions. For instance, Németh and colleagues [48] have demonstrated that lipopolysaccharide-stimulated BM-MSC release prostaglandin E2 which promotes the production of the anti-inflammatory cytokine IL-10 by macrophages. Similarly, by analyzing the secretome of BM-MSC cells with a new methodology, Milwid et al. [49] identified a previously unknown anti-inflammatory action of microfibrillar-associated protein 5 (MFAP5), which increased the production of IL-10 by lipopolysaccharide-stimulated monocytes and protected mice from a cytokine storm. Furthermore, extracellular vesicles, such as exosomes and microvesicles, are an important part of the BM-MSC secretome, as they can transfer functional mRNAs, miRNAs, proteins, and bioactive lipids with immunoregulatory and neuroprotective activities to target cells [45, 50, 51].

The anti-inflammatory role of transplanted BM-MSC and BM-MNC was evidenced by their ability to decrease the number of microglial cells/macrophages and neutrophils in the perihematomal region and to attenuate the expression of proinflammatory cytokines in the brain and/or plasma in animal models of ICH [24–26]. The anti-inflammatory protein TNF-*α* stimulated gene/protein 6 (TSG-6) probably mediated at least part of these effects, as suggested by Chen et al. [25], who found increased levels of TSG-6 in the brain of rats treated with an intravenous injection of BM-MSC 2 hours after the induction of ICH. Previous studies have shown that a large fraction of intravenously injected BM-MSC is retained in the lungs [52] and that the pulmonary passage induces them to secrete high levels of TSG-6, which contributes to the healing of the infarcted myocardium [53]. In addition, it was recently observed that the intravenous infusion of BM-MSC-derived extracellular vesicles, containing 4 ng of TSG-6, decreased the expression of the proinflammatory cytokine interleukin-1*β* in the brain and improved the cognitive function in a model of traumatic brain injury [54]. This is far lower than the dose of recombinant TSG-6 that promoted beneficial effects in this model of traumatic brain injury (50 *μ*g/mouse, intravenous) [55], suggesting that there may be other molecules in BM-MSC-derived extracellular vesicles that act synergistically with TSG-6 or that there are differences in the bioavailability of TSG-6 in these two conditions.

Studies using animal models of ischemic stroke have raised the possibility that umbilical cord blood- and bone marrow-derived cells could exert their anti-inflammatory action in the spleen [56, 57], where a large number of transplanted cells can be found for several days after transplantation [58–60]. Consistent with these findings, Suda et al. [26] observed that a significant number of intravenously administered BM-MNC migrated to the spleen in a rat model of ICH. However, it remains to be elucidated whether and how the interaction between transplanted cells and splenocytes contributes to the therapeutic effects of bone marrow-derived cells in this model.

Besides the anti-inflammatory action, BM-MSC and BM-MNC have been shown to exert neuroprotection in models of SAH and ICH. Many studies have found a reduced number of apoptotic cells in the brain [22–26, 33, 61] and/or a decrease in striatal tissue loss or lesion volume after the treatment [23, 27–29, 37, 61]. However, other studies showed no effects of the therapy on lesion volume [24, 35] or brain atrophy [62], despite the beneficial effect on functional recovery. A possible reason for this discrepancy is the fact that BM-MSC and BM-MNC can stimulate mechanisms of brain plasticity [62] and regeneration, including synaptogenesis [27–29], angiogenesis [24, 26], and neurogenesis [33, 35–37]. Moreover, the cell therapy can attenuate the blood-brain barrier dysfunction after ICH [25, 31]. In this regard, it has been shown that the pulmonary passage may be an important step for the protection of the blood-brain barrier by intravenously delivered BM-MSC. Menge et al. [63] showed that the interaction between pulmonary endothelial cells and BM-MSC induced the latter to secrete tissue inhibitor of matrix metalloproteinase-3 (TIMP3), thereby increasing the serum levels of TIMP3 in a model of traumatic brain injury. Silencing the expression of TIMP3 in BM-MSC abrogated the protective effects of the cell therapy in the blood-brain barrier [63]. Further work from this group has revealed that TIMP3 also has a direct neuroprotective role both* in vitro* and* in vivo* [64].

### 2.3. Quality of Preclinical Studies

Two recent meta-analyses concluded that the quality of original articles investigating the efficacy of stem cell therapies in animal models of ICH is still low [12, 13], as assessed by the Collaborative Approach to Meta-Analysis and Review of Animal Data from Experimental Stroke (CAMARADES) checklist [65]. For instance, studies using animals with relevant comorbidities (aged, hypertensive, or diabetic) are still rare. From the 19 studies included in our analysis, only 2 studies included animals with comorbidities. Wang et al. [31] subjected spontaneously hypertensive rats to the blood injection model of ICH and treated them with an intravenous injection of rat BM-MSC or vehicle. They showed that the cell therapy improved the recovery of behavioral function and increased the expression of the tight junction occludin in the blood-brain barrier of spontaneously hypertensive rats. However, the authors have not examined whether these effects were comparable to those reported in studies with normotensive rats. Another exception was the study of Suda and colleagues [26], who induced ICH in both young and aged rats. Animals received an intravenous injection of autologous BM-MNC or vehicle, 1 day after ICH. Both young and retired breeder aged rats benefited from the therapy, which decreased brain atrophy and edema and attenuated spatial learning deficits.

**Figure 3:**
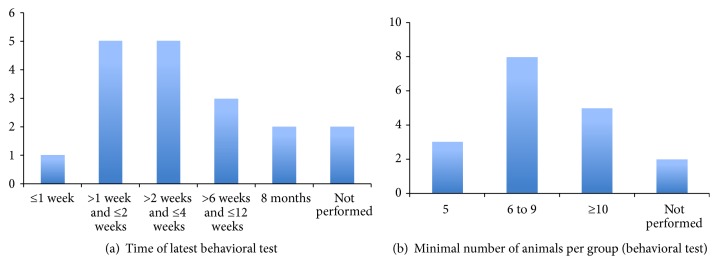
Charts summarizing the timing of the latest behavioral test (a) and the minimal number of animals per experimental group (b) in preclinical studies that have evaluated the therapeutic potential of bone marrow-derived cells for hemorrhagic stroke.

Other important parameters that are usually neglected or not informed by the authors are the control of body temperature during the surgeries, allocation concealment, blinded assessment of outcome, and sample size calculation [12, 13]. In an elegant review, MacLellan and colleagues [66] identified and discussed some of the limitations of the current preclinical research on ICH. Among these limitations, they highlighted that despite the fact that behavioral data collected at acute time points might be confounded by other factors, such as the presence of brain edema, the functional outcome is rarely assessed at protracted time points. They also found that the neurological deficit score (NDS), which includes a battery of subtests, was the most used behavioral method in models of ICH, whereas more sensitive tests were seldom performed. Indeed, among the 19 studies of ICH/SAH that we found, 13 had used NDS or modified versions of a neurological stroke scale (mNSS). Other sensorimotor behavioral tests that were used included the Rotarod (in 4 studies, 3 from the same group), the corner turn test (in 3 studies from the same group), the staircase test, and the adhesive removal test (used in 1 study each). Only one study also performed a cognitive test (Morris water maze task) [26] and two studies have not performed any behavioral assessments [33, 67]. We observed that the time of the last behavioral assessment varied widely, although almost all the studies have evaluated the animals for at least 2 weeks after the injury. In only one study, the behavioral analysis was restricted to the acute phase of stroke (Figure 3(a)) [25]. Similarly, the minimal number of animals per experimental group varied among the studies, ranging from 5 to 15 (Figure 3(b)).

Another criterion that is not included in the CAMARADES checklist is the inclusion of animals of both sexes with the separate analysis of data by sex, as recently recommended by the US National Institutes of Health (NIH) [68]. Among the 19 articles that we found, 7 studies have induced ICH only in females, 9 have subjected only male animals to ICH, and the 3 remaining studies have not informed the sex of the experimental animals (Figure 1(c)). Although there is conflicting evidence regarding the existence of sex-dependent differences in mortality and neurological outcome after ICH, numerous preclinical studies have indicated that gonadal hormones affect the pathophysiology of ICH [69], which in turn could affect the response to a given therapy [70].

We also noticed that while the cell dose and the infusion volume were reported in all studies, the infusion velocity was not informed. Both the cell dose and the infusion velocity can influence the occurrence of adverse events, such as the formation of microocclusions and the development of stroke, after the intra-arterial administration of BM-MSC in rats [71, 72]. These findings draw attention to the need for more studies on the long-term safety of cell therapies, such as the study by Donega et al. [73], who performed a systematic histopathological analysis of 38 organs, 14 months after the induction of neonatal hypoxia-ischemia and the intranasal transplantation of BM-MSC in mice. Their results showed no evidence of any adverse effects in the brain, nasal turbinates, or other peripheral organs.

## 3. Clinical Studies

We searched the PubMed/Medline database for original articles in English. We found 5 articles involving 5 different trials of cell therapies with a total of 188 treated patients with hemorrhagic stroke. Two trials were for basal ganglia hemorrhagic stroke and 3 for ischemic or hemorrhagic strokes (Table 2). One trial performed intravenous injections, 1 intrathecal injections, 2 intracerebral injections, and 1 intracerebral administration followed by intrathecal injections (Table 2).

Suarez-Monteagudo and coworkers [74] carried out a study with an IC injection of BM-MNC, which had 3 subjects with ischemic lesions in the cortex,* striatum,* or thalamus and 2 subjects with hemorrhagic lesions in the* striatum* or thalamus, between 3 and 8 years after the stroke. The patients with hemorrhagic lesions received a total of 1.7 × 10^7^ to 5.5 × 10^7^ BM-MNC by stereotactic implantation along different tracts next to the injury. The authors reported that no significant adverse events occurred in the 12-month follow-up. The authors described functional improvements at 12 months, with a decline in motor deficits assessed by Ashworth's Scale for Spasticity and the Medical Research Council Scale; an increase in the National Institutes of Health Stroke Scale (NIHSS), Barthel index (BI), and Scandinavian Stroke Scale; and improved locomotion and equilibrium, assessed by the Tinetti scale. Nevertheless, the absence of a control group, the small study size, and the unblinded evaluation do not permit conclusions concerning efficacy.

Li and collaborators [75] included subjects in a nonrandomized, phase I, single-blind study of BM-MNC therapy. Sixty patients received an intraparenchymal cell injection 5 to 7 days following basal ganglia hemorrhage, and 40 subjects were included in the control group. The total number of injected cells varied from 2.5 × 10^8^ to 2.3 × 10^9^ cells. The authors reported that after 6 months the NIHSS scores in the cell therapy group were significantly lower in comparison to the control group (*p* < 0.01), whereas the BI scores were higher (*p* < 0.01). Furthermore, the authors described that neurological and functional improvements occurred in 86.7% of the patients in the cell therapy group* versus* 42.5% in the control group (*p* = 0.001).

Bhasin and colleagues [76] carried out an unblinded nonrandomized trial with 4 subjects with ischemic middle cerebral artery (MCA) lesions and 2 with hemorrhagic MCA lesions, as well as 6 control subjects. Autologous BM-MSC were intravenously injected between 8 and 12 months after the stroke. Patients were evaluated through the Medical Research Council scale for muscle strength, the modified Ashworth Scale for spasticity, the Fugl-Meyer (FM) scale for motor recovery, and the modified BI. The authors reported that no therapy-related adverse effects occurred in the 6-month follow-up. Even though the analysis indicated an advance on the FM scale and modified BI after 2 and 6 months, no statistically significant difference was seen when comparing the BM-MSC-treated and control groups.

Sharma et al. [77] included patients with ischemic or hemorrhagic stroke in a phase 1, nonrandomized, open-label study. The authors stated that 30 patients were included but 6 were lost to follow-up. Of the remaining 24 patients, 10 had hemorrhagic and 14 ischemic lesions. Patients were treated 4 to 144 months after the stroke (mean 40.5 months) and received an intrathecal injection of 10^6^ cells multiplied by the body weight. Follow-up varied from 6 to 54 months (mean 30 months). The authors reported that 12 patients had improvement in ambulation, 10 in hand functions, 6 in standing balance, 9 in walking balance, and 10 patients in functional status. Three patients agreed to undergo PET with ^18^F-FDG before and after cell therapy. The authors reported an increase in brain metabolism after cell therapy in these patients.

Zhu and collaborators [78] carried out a clinical trial where 215 patients with ICH were included. Following surgical drainage and decompressive craniotomy, 114 patients agreed to receive the cell therapy while the remaining 101 patients participated as a control group. Three to six days after the surgery, bone marrow harvest was performed and a mean of 4 × 10^9^ BM-MNC was obtained. Half of the cell suspension was administered intracerebrally through indwelling drainage tubes that had been placed at the rim of the hematoma cavity. The other half of the cell suspension was separated for BM-MSC culture and a mean of 8.4 × 10^7^ cells was injected after 4 weeks by the intrathecal route. Four patients from the cell therapy group and 5 patients from the control group were lost to follow-up. The authors reported that there was no significant difference in demographic variables including age, gender, neurological findings, mean lesion volumes, and surgical methods between both groups. The authors reported that NIHSS scores and Rankin scale in the cell therapy group were lower and Barthel scores were higher than in the control group at 6 and 12 months. The authors stated that no transplantation-related adverse events occurred in the follow-up.

### 3.1. Ongoing Clinical Studies

A search in clinicaltrials.gov showed 3 ongoing registered studies, which are estimated to enroll up to 280 subjects with ischemic or hemorrhagic strokes (Table 3). However, none of the studies informed the proportion of ischemic to hemorrhagic stroke patients that will be included, and it is therefore not possible to know how many hemorrhagic stroke patients will be included. The intrathecal injection of autologous BM-MNC was chosen by two of the trials and the intravenous injection of autologous BM-MSC was chosen by the remaining trial (Table 3).

## 4. Discussion

Several interrogations remain concerning the potential use of cell therapies for hemorrhagic stroke. Amongst the key issues to be resolved is defining the optimal cell type to be transplanted. In 2013 the US Food and Drug Administration released its “Guidance for Industry: Preclinical Assessment of Investigational Cellular and Gene Therapy Products” stating that whenever feasible, the tentative product that will be delivered to the patients should be evaluated in the preclinical studies [79]. However, the delivery of human cells in preclinical studies may be challenging due to immune responses of animals, which may lead to rejection of the cells [79].

There are marked differences between the predominant cell types that have been chosen by preclinical and clinical studies for ICH. Four out of 5 clinical studies used BM-MNC while only 1 of the 19 preclinical studies studied this cell population. Moreover, while all clinical studies used autologous cells, either BM-MNC or BM-MSC, only 1 of the 19 preclinical studies used autologous cells. [26]. In the latter study, by Suda and collaborators, BM-MNC were collected 22 hours after ICH [26]. This approach was based on previous data demonstrating that the therapy with autologous BM-MNC was more effective if the cells were harvested 22 hours after the ischemic event, compared to cells harvested 24 hours before MCA occlusion in rats [80]. Such results suggest that stroke may prime BM-MNC (or perhaps a subpopulation of BM-MNC) towards a protective phenotype. Similar observations have been made in a model of acute intestinal infection with* Toxoplasma gondii*, where monocyte progenitors were primed for regulatory functions very early after infection, prior to their mobilization from bone marrow [81].

Notably, the transplantation of autologous poststroke BM-MNC is a feasible approach for the acute treatment of different types of stroke, since the BM-MNC fraction can be easily isolated in high yield and purity using density gradient media [20]. In contrast, the time required to expand BM-MSC in culture would not allow the transplantation of autologous BM-MSC in the acute phase of the injury, unless the cells had been banked for future use. Nevertheless, given their low immunogenicity, allogeneic MSC could be transplanted without the need for immunosuppressive drugs. This approach has been demonstrated in many preclinical and clinical studies for different diseases [20, 82]. For instance, Prochymal®, allogeneic culture-expanded adult human BM-MSC, was the first stem cell product to receive conditional market authorization from Health Canada for the treatment of acute graft-versus-host disease in pediatric patients who are unresponsive to corticosteroid [83]. Different trials using culture-expanded allogeneic bone marrow-derived cells have also been designed for ischemic stroke, including from US companies Athersys, SanBio, and Stemedica, but their final results have not yet been reported [84, 85].

It is possible to manipulate BM-MSC before injection in an attempt to increase their survival, to induce a desired phenotype or to modify their migratory capacity. This can be done by changing culture conditions (e.g., three-dimensional culturing), by genetic manipulation, or by exposing the cells to growth factors, immunomodulators, or low doses of toxic factors, for example [86]. Wei and coworkers have demonstrated that BM-MSC upregulate the expression of the growth factors brain-derived neurotrophic factor (BDNF), glial cell line-derived neurotrophic factor (GDNF), and vascular endothelial growth factor (VEGF) after 48 hours of culture under hypoxic conditions [87]. They also have observed that the intranasal injection of hypoxia-preconditioned BM-MSC (cultured for 24 hours under hypoxia) restored the expression of these same factors to near normal levels in the perihematomal region in mice [37].

Several lines of evidence have supported the notion that BM-MSC comprise a heterogeneous population of cells with distinct functions [88]. Therefore, the identification and enrichment of one or more subpopulations of BM-MSC with the desired functional attributes could represent a strategy to improve the therapeutic potential of BM-MSC. This was the approach used by Bao et al. [24], who investigated the effects of the intracerebral transplantation of fetal liver kinase- (Flk-) 1^+^ human BM-MSC, a subpopulation of BM-MSC that was the first stem cell product approved by the State Food and Drug Administration of China, in a rodent model of ICH. In sum, their results have shown that Flk-1^+^ BM-MSC improved the neurological recovery through anti-inflammatory, antiapoptotic, and proangiogenic effects.

Besides the intrapopulation heterogeneity, there may be differences in therapeutic efficacy among BM-MSC isolated from different donors. In an attempt to overcome this limitation, Prockop and colleagues have demonstrated that TSG-6 mRNA level could be used as a biomarker to predict the* in vivo* anti-inflammatory activity of human BM-MSC [89]. Accordingly, the establishment of potency assays is one of the main challenges for the development of cellular therapy products [90].

Finally, it has been demonstrated that MSC isolated from other tissues, such as the umbilical cord blood [91], umbilical cord tissue [92], or adipose tissue [93], can improve the behavioral recovery in animal models of ICH. However, given the intrinsic biological differences between MSC isolated from different tissues [94], it is still uncertain whether there are differences in their therapeutic potential.

The optimal cell dose also continues to be uncertain. The Stem Cell Therapies as an Emerging Paradigm in Stroke (STEPS) guidelines suggested dose conversion from preclinical studies based on body weight [95]. However, only one of the 5 clinical studies performed cell therapy based on body weight, and no preclinical study was used as rationale for dose conversion [77].

The use of imaging methods is another tool that can advance knowledge of numerous aspects, including cell migration and homing. Several methods can be employed to evaluate cell biodistribution. Although fluorescence and bioluminescence have been efficiently used to follow cells in animal studies for different neurological diseases, they have not been used in studies of cell therapies for hemorrhagic stroke and have limitations such as the restricted tissue penetration of light that does not allow their clinical use [96]. Superparamagnetic iron oxide nanoparticles (SPIOs), initially produced to identify hepatic tumors, may be used for exogenous cell labeling and tracking with MRI and has been used in a preclinical study with neural stem cell therapy for ICH [97]. However, SPIOs have restraints of exogenous contrasts, such as the chance of reduction with cell division. Furthermore, there are conflicting data on the impact of SPIOs in biological activities [98–101], and they have only been permitted for research studies.

For these reasons, nuclear medicine techniques such as PET and single photon emission computed tomography (SPECT) are important for the evaluation of cell migration and homing* in vivo* by indirect or direct assessments [40]. In indirect assessments, the uptake of ^18^F-FDG may be used to evaluate increased metabolism in the lesioned brain. One of the preclinical studies of BM-MSC therapy for ICH used ^18^F-FDG and indicated improved metabolism up to 13 weeks after cell transplantation [32]. One of the clinical studies also used ^18^F-FDG after intrathecal administration of autologous BM-MNC in 3 of the 24 patients with ischemic or hemorrhagic stroke and reported increased brain metabolism one year after cell therapy [77]. However, the description of ^18^F-FDG methods in the latter study was extremely limited and, therefore, does not allow any conclusive analysis. Moreover, the increase in ^18^F-FDG metabolism does not necessarily indicate the presence of transplanted cells or improved brain metabolism and may also occur if an inflammatory process takes place [102]. Cells may also be labeled with radiotracers such as Technetium-99m for direct cell tracking [40]. Although it has been proved feasible in preclinical and clinical studies for ischemic stroke [60, 103–108], it has not yet been employed in preclinical or clinical studies of cell therapies for hemorrhagic stroke.

## 5. Conclusion

Findings from preclinical reports indicate that cell transplantation may promote positive structural and functional effects in models of ICH and SAH. Nevertheless, the modes of action of these therapies are still under investigation. Clinical trials, most of them small, open-label, and nonrandomized, have been published and have reported that bone marrow-derived cell transplantation appears to be safe and feasible for hemorrhagic stroke. Nonetheless, there is a clear need for better preclinical and clinical studies, including randomized and blinded studies, to define the efficacy of cell transplantation for hemorrhagic stroke.

## Figures and Tables

**Table 1:** Preclinical studies.

Reference	Cell type	Route	Dose, infusion volume, rate, and timing	Animals	Model	Engraftment	Functional outcome	Cellular/molecular effects
Zhang et al., 2006 [30]	Rat BM-MSC	IA (carotid artery), IV (cervical vein), or ICV	2 × 10^6^ cells in 20 *µ*L saline, slowly injected on days 1, 3, 5, and 7 after ICH	Sprague-Dawley rats weighing 270–300 g	Injection of 0.5 U collagenase VII into the left *striatum* (caudate nucleus)	BrdU-labeled cells found around the bleeding focus, in the ipsilateral cortex and ipsilateral hippocampus (except in the IV group), on days 1, 3, 5 and 7 after injection	Improved (beam balance test), on days 1, 3, 5, and 7 after injection (IA and ICV groups only).	NA

Seyfried et al., 2006 [27]	Human BM-MSC	IV (tail vein)	3 × 10^6^, 5 × 10^6^, or 8 × 10^6^ cells in 1 mL PBS, slowly injected 1 day after ICH	Male Wistar rats weighing 270–320 g	Injection of 100 *µ*L autologous whole blood into the right *striatum*	Human cells were detected by the mAb 1281; cells were found in the injured region (14 days after ICH)	Improved in all groups (NSS and corner turn test), 7 and 14 days after ICH	Decreased striatal tissue loss (all tested doses); increased neurogenesis and synaptogenesis

Seyfried et al., 2008 [28]	Human BM-MSC	IA (internal carotid artery); IV injection of mannitol (1.5 g/kg) alone or 10 min before IA injection of BM-MSC	1 × 10^6^ cells in 100 *μ*L PBS, 1 day after ICH	Male Wistar rats weighing 270–320 g	Injection of 100 *µ*L autologous whole blood into the right *striatum*	Human cells were detected by the mAb 1281; more cells were found in the injured region in the IA BM-MSC + IV mannitol group (14 days after ICH)	Improved only in the IA BM-MSC + IV mannitol group (NSS and corner turn test), up to 14 days after ICH	Decreased striatal tissue loss; increased neurogenesis and synaptogenesis; improvements occurred only in the IA BM-MSC + IV mannitol group

Otero et al., 2010 [36]	Rat BM-MSC	IC (into the injured zone)	2 × 10^6^ cells in 10 *μ*L of saline, 3 days after ICH	Female Wistar rats weighing 200–250 g	Injection of 0.5 U collagenase IV into the *striatum*	BrdU-labeled cells and male (donor-derived) cells found near the lesion (1 month after transplantation)	Improved (Rotarod and mNSS), 3 and 4 weeks after ICH	Increased number of proliferating cells (Ki67+) in the SVZ

Seyfried et al., 2010 [29]	Rat BM-MSC	IV (tail vein)	0.5 × 10^6^ or 1 × 10^6^ cells in 1 mL PBS, 1 day after ICH	Female Wistar rats weighing 270–320 g	Injection of 0.1 cc of blood into the right *striatum*	Y chromosome-positive cells (donor-derived) found in the ipsilateral hemisphere (14 days after ICH)	Improved only in the group that received the higher dose (NSS and corner turn test), 7 and 14 days after ICH	Decreased striatal tissue loss (2 weeks after ICH); increased neurogenesis and synaptogenesis; improvements occurred only in the high-dose group

Yang et al., 2011 [61]	Rat BM-MSC or rat BM-MSC overexpressing GDNF	IC (into the right *striatum*)	5 × 10^5^ cells in 20 *μ*L saline, injected at 2 mL/min, 3 days after ICH	Wistar rats weighing 270–320 g	Injection of 0.25 U collagenase I into the right *striatum*	GFP-positive cells found mainly in the area surrounding the injection site (up to 2 weeks after transplantation)	Improved in both groups, but GDNF-BM-MSC improved further (mNSS), up to 2 weeks after ICH	Decreased lesion volume and decreased number of apoptotic cells in the *striatum* in both groups, but better results were obtained in the GDNF-MSC group; increased GDNF protein levels in the ipsilateral *striatum* (only in the GDNF-MSC group)

Otero et al., 2011 [34]	Rat BM-MSC	IC (into the injured zone)	5 × 10^6^ cells in 15 *μ*L of saline, 2 months after ICH	Adult female Wistar rats	Injection of 0.5 U collagenase IV into the *striatum*	Y chromosome-positive cells found in the vicinity of the lesion (six months after cell transplantation)	Improved (mNSS, Rotarod, and locomotor activity), up to 6 months after treatment	No change in the number of proliferating cells (Ki67+) in the SVZ

Feng et al., 2011 [32]	Human BM-MSC	IC (into 9 points near the hematoma)	1–5 × 10^6^ cells in 250 *µ*L of vehicle, 1 or 4 weeks after ICH	Male *Macaca fascicularis *monkeys weighing 4.2 ± 0.2 kg (4–6 y old)	Injection of 1.5 mL of blood between the right cortex and the basal ganglia (outside the right putamen)	NA	Improved in both the early and late treatment groups (neurologic deficit score), up to 8 weeks after treatment	Increased ^18^F-fluorodeoxyglucose uptake (better results were obtained in the early treatment group); increased microvessel density in the region surrounding the hematoma in both groups

Otero et al., 2012 [33]	Rat BM-MSC	IC (into the injured zone)	2 × 10^6^ cells in 15 *μ*L of saline, 2 hours after ICH	Female Wistar rats, weighing 200–250 g	Injection of 0.5 U collagenase IV into the *striatum*	Y chromosome-positive cells found in the vicinity of the lesion (up to 28 days after ICH)	NA	Increased number of proliferating cells (Ki67+) in the SVZ; decreased number of apoptotic cells in the lesion zone

Khalili et al., 2012 [22]	Rat BM-MSC	IV (tail vein)	3 × 10^6^ cells in 1 mL PBS, 1 day after SAH	Female Wistar rats weighing 275–300 g	Injection of 0.3 mL of blood into subarachnoid space	BrdU-positive cells were detected in the parietal lobe (14 days after SAH)	Improved (NSS), 14 days after SAH	Decreased number of apoptotic cells in the brain

Wang et al., 2012 [23]	Rat BM-MSC	IV (tail vein)	1 × 10^6^ cells in 1 mL PBS, 1 hour after ICH	Male Sprague-Dawley rats weighing 270–320 g (12 weeks old)	Injection of 0.4 U collagenase VII into the *striatum*	NA	Improved (mNSS), 7, 14, 21, and 28 days after ICH	Decreased hemorrhage volume; increased number of proliferating cells and decreased number of apoptotic cells in the perihematomal region; upregulated the expression of antiapoptotic molecules, G-CSF, and BDNF

Liang et al., 2013 [62]	Rat BM-MSC	IC (into 3 injection sites)	3 injections of 1 × 10^6^ cells in 10 *µ*L PBS each, 1 day after ICH	Male Wistar rats weighing 250–300 g (4 months old)	Injection of 0.8 U collagenase IV into the *striatum*	PKH26-labeled cells found in the lesion site, corpus callosum, and hippocampus (35 days after ICH)	Improved (modified limb placing test and vibrissae-elicited forelimb placing test), up to 28 days after ICH	No change in hemispheric atrophy; increased density of fibers crossing the midline; increased neuronal plasticity

Vaquero et al. 2013 [35]	Rat BM-MSC	IC (into the injured zone); one group received the cells embedded in a PRP scaffold	5 × 10^6^ cells in 30 *µ*L vehicle, injected over 5 min, 2 months after ICH	Female Wistar rats weighing 200–250 g	Injection of 0.5 U collagenase IV into the right *striatum*	Y chromosome-positive cells were found near the lesion and next to the SVZ; more cells were detected in the BM-MSC + scaffold group (6 months after cell transplantation)	Improved in both groups, but better results were obtained in the BM-MSC + scaffold group (Rotarod and locomotor activity test), up to six months after treatment	No significant change in lesion volume; increased number of proliferating cells (Ki67+) in the lesion zone (only in the BM-MSC + scaffold group) and in the SVZ (in both groups: BM-MSC alone or BM-MSC + scaffold)

Bao et al., 2013 [24]	Flk-1+ human BM-MSC	IC (into 3 injection sites)	2 × 10^5^ cells in 15 *μ*L of saline, 1 day after ICH	Male Sprague-Dawley rats weighing 190–210 g	Injection of 0.4 U collagenase VII into the *striatum*	Human cells were detected by the mAb 1281; cells were found close to the hemorrhagic boundary zone (55 days after transplantation)	Improved (mNSS), up to 56 days after ICH	Decreased brain water content; no significant change in hemorrhage volume; decreased number of microglia/macrophages and neutrophils in the hemorrhagic boundary; induced angiogenesis in the hemorrhagic boundary; decreased number of apoptotic cells and increased number of neurons in the hemorrhagic boundary; downregulated expression of several cytokines in the brain

Khalili et al., 2014 [67]	Rat BM-MSC	IV (tail vein)	3 × 10^6^ cells in 1 mL PBS, 1 day after SAH	Female Wistar rats weighing 275–300 g	Injection of 0.3 mL of blood into subarachnoid space	NA	NA	Improved ultrastructural morphology

Wang et al., 2015 [31]	Rat BM-MSC	IV (tail vein)	1 × 10^6^ cells in 100 *μ*L vehicle	Male spontaneously hypertensive rats	Injection of 50 *μ*L of blood into the *striatum*	PKH26-labeled cells were found in the brain (up to 42 days after ICH, although the number of cells decreased over time)	Improved (mNSS and modified limb placing test), up to 42 days after ICH	Increased expression of occludin in the brain

Sun et al., 2015 [37]	Hypoxia-preconditioned rat BM-MSC	Intranasal (30 min after the intranasal administration of 100 U hyaluronidase dissolved in PBS)	1 × 10^6^ cells in 100 *μ*L vehicle, 3 and 7 days after ICH	Male C57BL/6 mice weighing 25–28 g (8–10 weeks)	Injection of 0.15 U collagenase IV into the *striatum*	Hoechst 33342-labeled cells found in the olfactory bulb, ipsilateral cortex, perivascular spaces, and perihematomal regions (6 hours after transplantation)	Improved (mNSS, open field behavioral monitoring, Rotarod, and adhesive removal test), 14–21 days after ICH	Decreased tissue loss and ventricle enlargement; increased expression of GDNF, VEGF, and BDNF in the brain; increased SVZ neurogenesis

Chen et al., 2015 [25]	Rat BM-MSC	IV (jugular vein)	5 × 10^6^ in 200 *μ*L PBS injected over 10 min, 2 hours after ICH	Male Sprague-Dawley rats weighing 250–300 g	Injection of 0.5 U collagenase IV into the *striatum*	NA	Improved (mNSS), 3 days after ICH	Decreased brain water content; decreased number of apoptotic cells in the cortical hemorrhagic boundary; decreased number of microglia/macrophages and neutrophils in the brain; decreased blood-brain barrier dysfunction; decreased the expression of proinflammatory cytokines, MMP9, iNOS, and 3-nitrotyrosine in the brain; increased expression of anti-inflammatory cytokines and TSG-6 in the brain; suppressed activation of the NF-*κ*B signaling pathway

Suda et al., 2015 [26]	Autologous rat BM-MNC	IV (tail vein)	1 × 10^7^ cells/kg in 1 mL PBS infused over 5 min, 1 day after ICH	Male Long Evans rats weighing 275–325 g or retired breeder aged rats weighing 600–650 g	Injection of 70 *μ*L of blood into the *striatum*	Qtracker-labeled cells found in the brain, spleen, lungs, liver, and kidney (6 and 24 hours after transplantation)	Improved both in young and in aged rats (Staircase test, 28 days after ICH, and Morris water maze test, 4 weeks after ICH)	Decreased brain water content and brain atrophy both in young and in aged rats; stimulated angiogenesis and SVZ neurogenesis; decreased number of degenerating neurons, iNOS-positive cells, and neutrophils in the perihematomal area; decreased expression of HMGB1, MMP9, S100*β*, and aquaporin 4 in the brain; decreased levels of the proinflammatory cytokine interleukin-1*β* in the serum

BDNF: brain-derived neurotrophic factor; BM-MNC: bone marrow-derived mononuclear cells; BM-MSC: bone marrow-derived mesenchymal stem cells; BrdU: 5-bromo-2′-deoxyuridine; G-CSF: granulocyte-colony stimulating factor; GDNF: glial cell line-derived neurotrophic factor; GFAP: glial-fibrillary acidic protein; HMGB1: high mobility group box 1 protein; IA: intra-arterial; ICH: intracerebral hemorrhage; ICV: intracerebroventricular; iNOS: inducible nitric oxide synthase; IP: intraperitoneal; IV: intravenous; mAb: monoclonal antibody; MMP9: matrix metallopeptidase 9; mNSS: modified neurological stroke scale; NA: not available; NSS: neurological stroke scale; PRP: platelet-rich plasma; SAH: subarachnoid hemorrhage; SVZ: subventricular zone; TSG-6: tumor necrosis factor- (TNF-) stimulated gene 6 protein; VEGF: vascular endothelial growth factor.

**Table 2:** Trials with published clinical results.

Study reference/country	Cell type	Route	Study design	Stroke type	Age range (mean)	Time range from stroke to cell therapy	Number of treated patients with hemorrhagic stroke/total number of treated patients	Number of controls patients with hemorrhagic stroke/total number of control patients	Number of transplanted cells	Infusion volume, duration, and rate	Follow-up
Suarez-Monteagudo et al., 2009/Cuba [74]	Autologous BM-MNC	IC	Case series, nonrandomized, open-label	Ischemic or hemorrhagic in thalamus, basal ganglia, or cortex	41–64 (mean 51.4)	3 to 8 years (mean 5 years)	2/5	None	1.7 × 10^7^ to 5.5 × 10^7^ (mean 3.6 × 10^7^)	8 seeds of 2.5 *µ*L	12 months

Bhasin et al., 2011/India [76]	Autologous BM-MSC	IV	Case control, nonrandomized, open-label	MCA ischemic or hemorrhagic stroke	Mean 45	Mean 9.6 months	2/6	1/6	5 × 10^7^ to 6 × 10^7^	250 mL in 3 h (1.4 mL/min)	6 months

Li et al., 2013/China [75]	Autologous BM-MNC	IC	Phase 1, nonrandomized, single-blind	Basal ganglia hemorrhagic stroke	39–74 (mean 56.3)	5 to 7 days (mean 5.9 days)	60/60	40/40	2.5 × 10^8^ to 2.3 × 10^9^ (median 1.3 × 10^9^)	3.5 mL; duration not specified	6 months

Sharma et al., 2014/India [77]	Autologous BM-MNC	IT	Phase 1, nonrandomized, open-label	Ischemic or hemorrhagic stroke	27–79 (mean 57)	4 to 144 months (mean 40.5)	10/24	None	Body weight × 10^6^	Not specified	6 to 54 months (mean 30)

Zhu et al., 2015/China [78]	Autologous BM-MNC (IC) and BM-MSC (IT)	IC and IT	Not specified	Basal ganglia hemorrhagic stroke	32–75 (mean 57.2)	IC injection (BM-MNC): 3.01 to 6.89 days (mean 5.5)/IT injection (BM-MSC) after 4 weeks	114/114	96/96	Mean 2 × 10^9^ BM-MNCs/mean 8.4 × 10^7^ BM-MSC	5 mL for IC injection	12 months

IC: intracerebral; IV: intravenous; IT: intrathecal; BM-MNC: bone marrow mononuclear cells; BM-MSC: bone marrow-derived mesenchymal stem cells; MCA: middle cerebral artery.

**Table 3:** Ongoing trials registered in clinicaltrials.gov.

Trial identifier/country	Cell type	Route	Study design	Type of stroke	Age range	Time from onset to transplantation	Number of stroke patients to be included (controls)	Number of injected cells	Started	Status
NCT02245698/India	Autologous BM-MNC	IT	Phase 1, nonrandomized, open-label	Ischemic or hemorrhagic stroke	18–80	Not specified	200 (no controls)	Not specified	12/2008	Recruiting

NCT01714167/China	Autologous BM-MSC	IC	Phase 1, nonrandomized, open-label	Ischemic or hemorrhagic stroke	40–70	3–60 months	30 (controls not specified)	2 × 10^6^ to 4 × 10^6^	06/2012	Recruiting

NCT01832428/India	Autologous BM-MNC	IT	Phase 1–2, nonrandomized, open-label	Ischemic or hemorrhagic stroke	18–70	Not specified	50 (no controls)	1 × 10^8^ (3 doses with one-week interval)	09/2014	Recruiting

IC: intracerebral; IT: intrathecal; BM-MNC: bone marrow mononuclear cells; BM-MSC: bone marrow-derived mesenchymal stem cells.
